# From Theatre to Intensive Care: A Narrative Review of Life-Threatening Complications in Gynaecological Laparoscopy and Hysteroscopy

**DOI:** 10.7759/cureus.95649

**Published:** 2025-10-29

**Authors:** Mohamed Hemdan, Mahmoud Helaly, Hassan Idris, Mohamed Alosta

**Affiliations:** 1 Obstetrics and Gynaecology, Manchester University NHS Foundation Trust, Manchester, GBR; 2 Critical Care Medicine, Manchester University NHS Foundation Trust, Manchester, GBR

**Keywords:** anaesthetic management, fluid overload, gas embolism, gynaecological laparoscopy, hysteroscopy, intensive care, life-threatening complications

## Abstract

This narrative review examines life-threatening complications of gynaecological laparoscopy and hysteroscopy, synthesising their historical evolution, complication classification, and clinical implications. A structured search of PubMed and EMBASE (from January 2000 to August 2025), supplemented by guidance from international surgical and anaesthetic societies, identifies studies reporting catastrophic events, including major haemorrhage, visceral and vascular injury, fluid overload, electrolyte imbalance, gas embolism, thromboembolism, and pneumoperitoneum-related cardiopulmonary instability. Because of heterogeneity and frequent case-based evidence, the PRISMA (Preferred Reporting Items for Systematic Reviews and Meta-Analyses) methodology and meta-analysis are not applied. The review explores how patient, procedure, and operator factors can cause rapid deterioration from intraoperative incident to critical illness requiring intensive care. It summarises prevention and management strategies from current guidelines, emphasising careful patient selection, robust perioperative monitoring, anaesthetic vigilance, protocolised escalation pathways, and simulation-based team training, particularly relevant to outpatient hysteroscopy. Timely recognition and effective multidisciplinary coordination are key factors in ensuring patient survival and recovery. The review also discusses technological innovations, such as fluorescence-guided imaging, safer distension and insufflation systems, and predictive analytics, combined with the requirement for institutional adoption of safety culture, structured escalation frameworks, and continuous education. Persistent challenges include under-reporting, variability in definitions, and scarce long-term outcome data, which obscure true incidence and limit comparative analyses. By combining historical and recent evidence within a pragmatic clinical framework, this review provides clinicians with an integrated approach to anticipate, recognise, and manage life-threatening complications effectively. Future progress depends on standardised reporting, multicentre collaboration, and the evaluation of emerging monitoring technologies to ensure that minimally invasive gynaecology continues to advance while safeguarding patient safety.

## Introduction and background

Minimally invasive gynaecological surgery, particularly laparoscopy and hysteroscopy, has become a cornerstone of modern clinical practice, replacing many open surgeries with safer and more efficient alternatives. Both techniques are related to faster recovery times, less postoperative pain, shorter hospital stays, and better patient satisfaction than laparotomy [1-5]. Over the past two decades, advances in imaging, surgical technology, distension and insufflation media, and anaesthetic care have enhanced outcomes and expanded the scope of minimally invasive surgery [6-8].

However, alongside these massive benefits lies the reality that both laparoscopy and hysteroscopy can give rise to rare but potentially catastrophic complications. Although rare, these events can rapidly progress from minor intraoperative issues to life-threatening emergencies, necessitating early recognition and multidisciplinary coordination [9-11].

This challenge is particularly relevant in outpatient and ambulatory settings, where escalation pathways may be limited, underscoring the importance of anticipating adverse events, maintaining robust perioperative monitoring, and ensuring close collaboration between surgical, anaesthetic, and critical care teams [12-14].

The range of possible complications differs between the two procedures, but they can be equally serious. In hysteroscopy, the use of distension media creates unique physiological challenges, and complications, such as fluid overload, electrolyte imbalance, and sudden cardiovascular compromise, have been widely documented [2,3,7,15]. Mechanical complications, including uterine perforation, haemorrhage, and gas embolism, although uncommon, can escalate quickly and lead to catastrophic outcomes if not identified early and managed [16,17]. In laparoscopy, visceral or vascular injury, pneumoperitoneum-related cardiopulmonary instability, and massive haemorrhage remain key causes of life-threatening deterioration [4,5,18,19]. These complications can happen even in low-risk procedures, showing that minimally invasive surgery still carries a limited safety margin.

Furthermore, the increasing shift towards office hysteroscopy in an outpatient setting and laparoscopy with same-day discharge has expanded the spectrum of potential risk, creating new challenges in early recognition and referral when emergencies arise outside the operating theatre [3,8,12,20]. Such developments highlight the need for structured escalation frameworks, simulation-based training, digital monitoring systems, and adherence to professional guidance such as the Royal College of Obstetricians and Gynaecologists’ Green-top Guideline No. 59, which emphasises patient selection, preoperative risk assessment, and structured intraoperative monitoring protocols [8,14,21].

The objective of this review is to critically appraise the mechanisms, incidence patterns, prevention and recognition strategies, and intensive-care outcomes of life-threatening events in gynaecological laparoscopy and hysteroscopy. In addressing this objective, the review synthesises evidence on the pathophysiology and risk factors underlying catastrophic complications, evaluates warning signs and preventive interventions, and examines multidisciplinary management strategies. It further highlights the contribution of technological innovations, artificial intelligence-assisted analysis, and team simulation in enhancing intraoperative safety and escalation response [6,10,18,19,21].

By combining historical and recent literature, this review situates life-threatening complications within the broader trajectory of surgical progress and patient-safety innovation, aiming to ensure that minimally invasive gynaecology continues to evolve but never at the expense of patient safety [5,8,19,21].

## Review

Methods

This review was designed as a narrative synthesis supported by a structured literature search of peer-reviewed studies and professional guidelines. Because of data heterogeneity and the inclusion of case reports describing rare events, a formal PRISMA (Preferred Reporting Items for Systematic Reviews and Meta-Analyses)-based systematic review or meta-analysis was not undertaken. Searches were conducted in PubMed and EMBASE for publications in English from January 2000 to August 2025, covering the period of widespread adoption of minimally invasive gynaecological surgery. Additional sources included professional guidance from the Royal College of Obstetricians and Gynaecologists (RCOG), the American Association of Gynaecologic Laparoscopists (AAGL), the International Society for Gynaecologic Endoscopy (ISGE), the Society of American Gastrointestinal and Endoscopic Surgeons (SAGES), the European Society of Anaesthesiology and Intensive Care (ESAIC), and other related surgical and anaesthetic societies.

Search terms combined procedure, complication, and outcome concepts, including laparoscopy, hysteroscopy, complications, vascular injury, gas embolism, fluid overload, hyponatraemia, ICU, critical care, and mortality. Boolean operators and Medical Subject Headings (MeSH) were used to refine results, and reference lists of relevant articles were hand-searched to capture additional sources.

Publications were included if they reported life-threatening or intensive-care-level complications associated with gynaecological laparoscopy or hysteroscopy, or if they provided mechanistic or physiological insights applicable to such events. Reports limited to minor complications, non-gynaecological procedures, or experimental studies were excluded unless they offered transferable principles (for example, endoscopic gas embolism or effects of pneumoperitoneum on cardiopulmonary function). Preference was given to systematic reviews, multicentre cohort studies, clinical guidelines, and well-documented case series, while case reports were selectively included to illustrate rare mechanisms or ICU outcomes.

Evidence was synthesised thematically into six domains reflecting the structure of this paper: historical evolution, classification of complications, life-threatening events, risk factors and predictors, prevention and management strategies, and patient outcomes. No formal quantitative pooling or risk-of-bias scoring was performed, and findings are presented narratively to contextualise patterns and highlight gaps in the current literature. Table 1 shows a brief summary of the search process and framework.

**Table 1:** Summary of Literature Search and Framework RCOG, Royal College of Obstetricians and Gynaecologists; AAGL, American Association of Gynaecologic Laparoscopists; ISGE, International Society for Gynaecologic Endoscopy; SAGES, Society of American Gastrointestinal and Endoscopic Surgeons; ESAIC, European Society of Anaesthesiology and Intensive Care.

Parameter	Description
Databases searched	PubMed and EMBASE
Search period	January 2000-August 2025
Language restriction	English only
Search strategy	Combined procedure, complication, and outcome terms using Boolean operators and MeSH headings: laparoscopy, hysteroscopy, complications, vascular injury, gas embolism, fluid overload, hyponatraemia, ICU, critical care, mortality
Additional sources	Professional guidelines and statements from RCOG, AAGL, ISGE, SAGES, ESAIC, and other related surgical and anaesthetic societies
Inclusion criteria	Studies and reports describing life-threatening or ICU-level complications of gynaecological laparoscopy or hysteroscopy; mechanistic or physiological insights relevant to these events
Exclusion criteria	Reports limited to minor complications, non-gynaecological procedures, or experimental/non-clinical studies
Study types	Systematic reviews, multicentre cohort studies, professional guidelines, and well-documented case series; selected case reports illustrating rare mechanisms or ICU outcomes
Data synthesis approach	Narrative synthesis organised into six domains: historical evolution, classification, life-threatening events, risk factors, prevention/management, and outcomes; no quantitative pooling or risk-of-bias scoring performed

Narrative review

Historical Evolution of Laparoscopy and Hysteroscopy

The evolution of gynaecological laparoscopy and hysteroscopy is one example of changes seen in surgical practice, where the development of new technologies occurred at the same time anaesthetic techniques improved until safer, less invasive alternatives to open surgery were developed. Initially, laparoscopy was limited by inadequate instrumentation, rudimentary optics, and concerns over anaesthetic safety, restricting its role to diagnostic applications. Over time, advances in surgical tools, energy systems, and distension media enabled clinicians to extend the scope of both laparoscopy and hysteroscopy to therapeutic interventions. These improvements not only shortened recovery times and reduced morbidity but also changed the patient experience, offering once unimaginable alternatives [1,7,11]. Importantly, the growing sophistication of anaesthesia, particularly in tailoring approaches for different populations, contributed significantly to the expansion of these procedures. As Roddy et al. [22] emphasise, continuous refinement of anaesthetic strategies tailored to patient physiology, comorbidities, and procedure complexity was pivotal in reducing intraoperative instability and postoperative critical-care admissions in gynaecological laparoscopy. These are examples of the joint evolution of anaesthetic and surgical practice that underpin for safety of minimally invasive surgery.

Similarly, hysteroscopy has been shaped by technological and technical progress. Early hysteroscopic procedures were hampered by poor visibility and complications linked to distension media. The refinement of distending solutions and development of high-definition fibre-optic systems provided clearer operative fields and reduced complication rates [6,23]. With the development in the use of hysteroscopy for myomectomy, adhesiolysis, and other therapeutic applications, management of intrauterine disorders was greatly enhanced by hysteroscopic technology, but at the same time introduced a number of new operative complications, including intrauterine adhesions. Systemic inflammatory responses and endometrial injury may promote significant adhesion formation even after apparently uncomplicated hysteroscopic procedures, highlighting that the advancement of hysteroscopy continues to require balancing therapeutic innovation with the careful management of emerging risks [24]. This historical pattern demonstrates how innovation, while expanding therapeutic horizons, has consistently introduced new dimensions of risk requiring careful evaluation and governance [25,26].

The adoption of laparoscopy in routine gynaecological practice has been driven by both technological advances and the ongoing need to ensure procedural safety in varied clinical settings. During the COVID-19 pandemic, international societies emphasised adapting minimally invasive techniques to preserve patient safety and minimise perioperative risk without compromising surgical standards [12,27]. This period reinforced the importance of strong perioperative protocols, effective team communication, and thorough contingency planning, all essential for preventing and managing life-threatening complications in gynaecological endoscopy [12,27,28].

Refinement of surgical indications also forms a critical aspect of the historical evolution of these procedures. With accumulating evidence, minimally invasive techniques began to replace laparotomy in increasingly complex scenarios such as ectopic pregnancy, endometriosis, and gynaecological oncology [29]. More recently, laparoscopy has been applied to complex conditions such as caesarean scar pregnancy, demonstrating its feasibility and safety even in high-risk situations. These advances show how minimally invasive surgery has progressed from a simple diagnostic technique to a versatile therapeutic approach capable of managing serious complications [18]. However, the extension of these techniques into higher-risk settings has introduced additional perioperative challenges, such as bleeding, organ injury, and the potential need for intensive care support, emphasising the importance of ongoing vigilance and institutional readiness [16,18].

The role of educational and institutional initiatives in minimising catastrophic complications in minimally invasive gynaecology is crucial. Structured simulation and certification programmes from professional bodies such as the ISGE, AAGL, and RCOG have demonstrated measurable improvements in intraoperative crisis management, escalation response, and postoperative outcomes [9,20,21]. These initiatives highlight that advanced technology must be supported by validated operator competence and a sustained institutional safety culture to prevent progression from intraoperative incidents to critical-care admission [10,21].

Classification of Complications in Minimally Invasive Gynaecology

Levy and Tsaltas [30] stressed that despite the majority of complications in benign laparoscopic procedures being minor, a small but significant risk of life-threatening events persists, demanding constant vigilance. Complications in minimally invasive gynaecology can be broadly categorised according to their mechanical, haemorrhagic, systemic, and anaesthetic origins. Historically, these were described mainly in isolated case reports, but the evolution of evidence-based practice has encouraged a more structured classification system that distinguishes between the mechanism, severity, and systemic consequences of complications [31,32]. Not only does this framework allow clinicians a way to anticipate adverse events, but it also underscores the continuum from minor to catastrophic outcomes. Such a spectrum highlights the paradox of minimally invasive gynaecology: despite its safety and efficacy, even minor technical lapses can have serious consequences [32].

Mechanical complications continue to represent a significant proportion of adverse events, most commonly involving injury to the uterus, bowel, bladder, or ureters. Such incidents often occur from blind entry, excessive tissue traction, or impaired visualisation in anatomically distorted anatomy. A related but more catastrophic outcome is uterine rupture, which, although rare, has been associated with prior hysteroscopic septum resection. Although uncommon, uterine rupture in subsequent pregnancies highlights the long-term consequences of previous intrauterine surgery. These mechanical injuries may vary in severity, ranging from minor events requiring simple repair to major complications necessitating laparotomy or postoperative intensive care [33].

Haemorrhagic complications represent another critical category, posing immediate surgical and systemic challenges. Intraoperative bleeding may result from vascular injury during laparoscopy or uterine perforation during hysteroscopy, and can rapidly escalate if not promptly identified and controlled [4,5,34]. In certain cases, bleeding may be concealed as haemoperitoneum, delaying diagnosis and increasing the risk of circulatory collapse. Even with meticulous technique, haemorrhage may be unavoidable in procedures complicated by adhesions or vascular anomalies. These events bridge the gap between routine surgical issues and catastrophic complications, reminding clinicians that minimally invasive surgery does not eliminate the fundamental risks of operative trauma [5].

Systemic and anaesthetic-related complications are less common but potentially more fatal. Pneumoperitoneum, an essential component of laparoscopic surgery, has been associated with profound cardiovascular disturbances, including vagally mediated bradycardia and cardiac arrest. Petker and Ahmed [35] reported an asystolic cardiac arrest triggered by vagal stimulation from carbon dioxide insufflation during laparoscopic gynaecological surgery. Likewise, fluid overload and electrolyte imbalance during hysteroscopy can cause severe neurological or cardiovascular sequelae [13]. These systemic complications demonstrate the complexity that exists between surgical approach, anaesthetic management, and patient physiology, further endorsing a cohesive multidisciplinary vigilance [10].

**Figure 1:**
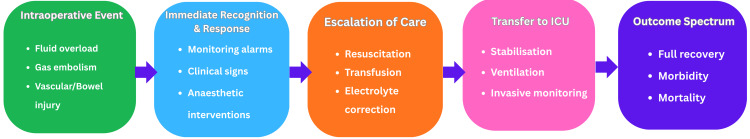
From Theatre to Intensive Care: Pathway of Life-Threatening Complications in Gynaecological Laparoscopy and Hysteroscopy Created by the authors.

Finally, the classification of complications in minimally invasive gynaecology has evolved to include contextual and systemic factors. During the COVID-19 pandemic, societies such as the SASGE and ISGE published guidance urging modifications to surgical practice to mitigate viral transmission risks while maintaining patient safety [12,36]. This broader view acknowledges that adverse events extend beyond patient physiology to encompass healthcare system and provider safety, especially in high-pressure environments. Consequently, modern classification now integrates mechanical, haemorrhagic, systemic, and situational factors, reflecting the complex and dynamic nature of risk in minimally invasive surgery [37]. The escalation pathway from intraoperative complication to intensive-care admission is summarised in Figure 1, outlining the critical transition points and physiological mechanisms underpinning life-threatening deterioration.

Life-Threatening Complications in Hysteroscopy

Hysteroscopy has established itself as a safe and minimally invasive approach for diagnosing and treating intrauterine pathologies; however, rare but life-threatening complications continue to be reported. One of these complications occurs due to excessive absorption of distension media, which can result in fluid overload and significant electrolyte disturbances. Excessive intravasation of hypotonic or electrolyte-free solutions can trigger hyponatraemia, pulmonary oedema, and cerebral oedema, which may progress rapidly to cardiovascular collapse if unrecognised. The risk increases with prolonged procedures, high intrauterine pressures, or large volumes of distension fluid, highlighting the importance of meticulous fluid balance monitoring and intrauterine pressure control [6,7,13,21]. Although hysteroscopy is less invasive than laparotomy, its systemic risks, particularly those related to intravascular absorption, can occasionally exceed those of open procedures, underscoring the need for anaesthetic vigilance and structured peri-operative monitoring [10].

Another well-documented but rare complication is venous gas embolism (VGE), resulting from gas entering the venous circulation during hysteroscopic procedures. Vilos et al. [14] reported five cases of VGE during endometrial ablation, demonstrating that even standard procedures can carry catastrophic potential. Gas embolism causes acute right-heart strain, hypoxaemia, and cardiovascular collapse, often presenting abruptly and requiring immediate recognition. While improvements in technique and anaesthetic monitoring have reduced its incidence, reported cases persist even in modern practice [14,21]. This underlines the need for continuous communication between the surgical and anaesthetic teams and adherence to preventive measures such as avoiding overpressurisation of the uterine cavity and ensuring adequate gas evacuation before instrumentation [8,14,21].

Uterine perforation is another significant risk with potentially fatal outcomes. Minor perforations may heal spontaneously, but major injuries can result in haemorrhage, visceral trauma, and infection, requiring laparotomy or intensive care admission [15,33]. Perforation risk increases with difficult uterine anatomy, cervical stenosis, or distorted cavities from fibroids or previous surgery. Krentel et al. [15] demonstrated that emerging diagnostic tools, such as fluorescence-guided laparoscopy, may improve early detection of uterine niche defects and related injuries, providing an avenue for prompt intervention. Despite technological advances, however, no method fully eliminates the risk, reinforcing the importance of intraoperative caution and postoperative vigilance [6,15,25,26].

Complications in hysteroscopy are also influenced by prior uterine surgery and obstetric history. For instance, hysteroscopic evaluation in women with caesarean scar defects or previous uterine interventions carries higher risks of haemorrhage or uterine rupture. Laparoscopic management of caesarean scar pregnancies offers a safer alternative in cases where unrecognised vascular invasion could otherwise lead to catastrophic haemorrhage during hysteroscopic intervention [18,38]. These cases illustrate that hysteroscopic complications cannot always be viewed in isolation but are shaped by patient-specific factors such as uterine scarring, comorbidities, and surgical history [8,18,21].

Ultimately, the rare but severe complications of hysteroscopy, fluid overload, gas embolism, and perforation demonstrate the delicate balance between minimally invasive benefits and systemic risk. Preventive strategies include limiting intrauterine pressure, meticulous fluid monitoring, and use of modern distension systems with automated safety cut-offs [7,13,21]. Anaesthetic preparedness, skilled surgical technique, and institutional escalation protocols are equally essential to ensure timely recognition and management. The literature consistently emphasises that the continued success of hysteroscopy depends not only on technological innovation but also on unrelenting clinical vigilance, ensuring that the journey from theatre to intensive care remains a preventable rarity in contemporary practice [6,10,21].

Life-Threatening Complications in Laparoscopy

Laparoscopic surgery, though widely regarded for its faster recovery and reduced postoperative morbidity, can also precipitate rare but life-threatening complications. The most feared is major vascular injury, typically occurring during trocar insertion or instrument manipulation. Injury to large vessels, such as the aorta, vena cava, or iliac arteries or veins, may result in sudden, rapid haemorrhage, requiring prompt recognition and immediate conversion to laparotomy for repair [4,16,34]. Although the reported incidence of such events is low, mortality can reach up to 15% in some series [16]. Risk factors include prior abdominal surgery, obesity, and anatomical variants, all reinforcing the need for adherence to optical entry techniques, preoperative imaging, and preparedness for emergency conversion [4,10,16].

Bowel perforation remains another critical complication. It may arise from blind trocar entry, thermal injury, or instrument traction, especially when energy devices are used near adherent bowel loops [4,5]. Delayed recognition substantially increases morbidity and mortality due to peritonitis and sepsis. Early detection requires high intraoperative suspicion and postoperative monitoring for subtle signs such as tachycardia or peritoneal irritation. Although laparoscopic magnification offers visual advantages, bowel injury remains a key contributor to ICU admission in minimal-access gynaecology [5,11].

Severe haemorrhage can result from vascular injury or tissue dissection. Despite smaller incisions, bleeding during laparoscopy may obscure the field and cause rapid haemodynamic instability. Studies highlight that perioperative venous thromboembolism and bleeding remain significant complications even in non-cancer gynaecologic operations, necessitating balanced use of thromboprophylaxis and haemostatic measures [4,16,21,34]. Adequate access to blood products, readiness for open conversion, and structured damage-control strategies are vital for preventing catastrophic outcomes.

Anaesthetic and physiological complications also pose unique risks. Creation of pneumoperitoneum alters venous return and diaphragmatic excursion, potentially causing arrhythmia, myocardial ischaemia, or respiratory compromise, particularly in patients with pre-existing comorbidities [17,35]. Continuous monitoring of end-tidal CO₂ and invasive haemodynamics is recommended in high-risk cases. Reports of vagally mediated bradycardia and asystole underscore the need for close teamwork between surgeons and anaesthetists [10,17,35].

Finally, thromboembolic events, including deep vein thrombosis and pulmonary embolism, are still recognised postoperative risks, even though laparoscopic surgery generally allows faster recovery and earlier mobilisation. Prolonged procedures, obesity, and inadequate prophylaxis increase risk, supporting guideline-driven preventive strategies [4,9,34]. As Madhok et al. [4] note, perioperative risk reduction through preoperative screening, pharmacologic prophylaxis, and intraoperative positioning remains as crucial as technical skill. A summary of life-threatening complications in gynaecologic hysteroscopy and laparoscopy is presented in Table 2, outlining key mechanisms, incidence ranges, and clinical consequences.

**Table 2:** Major Life-Threatening Complications in Gynaecological Laparoscopy and Hysteroscopy

Complication	Procedure	Mechanism	Reported Incidence	Clinical Consequences	References
Fluid overload/hyponatraemia	Hysteroscopy	Excessive absorption of distension media	~0.1-0.2 % (in modern practice)	Seizures, cerebral oedema, cardiovascular collapse	[6,7,13,21]
Gas embolism	Laparoscopy and hysteroscopy	Entry of CO₂ or distension gas into venous circulation	Rare; clinically significant events reported up to ~0.1 % (hysteroscopy); very rare in benign laparoscopy	Cardiovascular collapse, cardiac arrest	[11,14,21]
Major haemorrhage	Laparoscopy and hysteroscopy	Vascular injury, uterine perforation	~0.1-0.3 % (hysteroscopy); ~0.1-0.5% (laparoscopy, higher in complex cases)	Hypovolemic shock, need for transfusion/ICU	[4,16,21,34]
Bowel/visceral injury	Laparoscopy	Trocar or electrosurgical trauma	~0.1-0.3 % (overall; higher in complex hysterectomy/severe endometriosis)	Peritonitis, sepsis, multiorgan failure	[4,5,11,16,34]
Electrolyte imbalance	Hysteroscopy	Hypotonic media absorption	~0.1-0.2% (in modern practice)	Arrhythmias, neurological deterioration	[6,7,13,21]

To complement existing summaries of individual complications, Table 3 presents a comparative overview of the reported incidence ranges of major life-threatening events in gynaecologic laparoscopy and hysteroscopy, consolidating data from contemporary reviews.

**Table 3:** Comparative Incidence of Major Life-Threatening Complications in Gynaecologic Laparoscopy and Hysteroscopy “—” indicates that no reliable or procedure-specific incidence data were reported. VTE, venous thromboembolism; PE, pulmonary embolism.

Complication	Hysteroscopy – Reported Incidence	Laparoscopy – Reported Incidence	References
Major haemorrhage	~0.1-0.3% (higher with operative myomectomy)	~0.1-0.5% (procedure-dependent; higher in complex cases)	[4,11,16,19, 34]
Venous gas embolism	Rare; clinically significant events up to ~0.1%	Very rare in benign gyn laparoscopy	[11,14,21]
Visceral/bowel injury	Exceptionally uncommon in diagnostic/office settings	~0.1-0.3% overall; higher with complex hysterectomy or severe endometriosis	[4,5,11,16,34]
Fluid overload/hyponatraemia; electrolyte imbalance	~0.1-0.2% in modern practice; risk ↑ with hypotonic media, high pressure, long cases	—	[6,7,8,13,23]
Thromboembolism (VTE/PE)	Very rare in office/short procedures (<0.1%)	~0.2-0.3% in benign gyn surgery with modern prophylaxis	[4,19,34,39]

Risk Factors and Predictors of Severe Adverse Events

Understanding the determinants of life-threatening complications in minimally invasive gynaecology is essential to strengthen perioperative safety. These determinants can be broadly grouped into patient-, procedure-, and operator-related factors.

Patient-related risks include advanced age, cardiovascular disease, diabetes, obesity, and immunocompromise, all of which predispose to poor haemodynamic tolerance and delayed recovery. Pre-existing cardiac or pulmonary disease increases the likelihood of intraoperative instability triggered by pneumoperitoneum, fluid overload, or anaesthetic stress, highlighting the need for detailed preoperative assessment and tailored anaesthetic management [4,9,17,39].

Procedure-related factors encompass case complexity, duration, intra-cavity pressure, and fluid or gas exposure. Prolonged laparoscopy or hysteroscopy increases absorption of distension media, risk of gas embolism, and cardiopulmonary compromise [6,7,13,17,21]. Safety-optimised instrumentation, pressure-regulated systems, and limiting operative time are proven to reduce morbidity [8,21,36]. Moreover, inadequate thromboprophylaxis or emergency conversion to laparotomy adds further systemic risk [4,16,34].

Operator and team factors strongly influence the outcome. Surgeons in the early phase of their learning curve experience higher rates of mechanical injury, haemorrhage, and conversion [9,20]. Simulation-based and credentialing programmes endorsed by ESGE, AAGL, and RCOG have demonstrated measurable improvements in technical performance and crisis response [20,21].

Emerging evidence also points to biological and digital predictors of adverse events. Perioperative inflammatory biomarkers such as interleukin-6 and the neutrophil-to-lymphocyte ratio, coupled with machine-learning risk models, may improve early identification of patients at risk of deterioration [40-42]. Integration of such predictive analytics into perioperative monitoring could enable real-time risk adjustment and earlier intervention, marking a shift from reactive to anticipatory patient safety.

Large multicentre and registry-based studies have improved the understanding of adverse event rates in minimally invasive gynaecology. National surveillance systems, including the Medicare Patient Safety Monitoring System, offer important data on perioperative complications and reintervention rates in benign gynaecologic surgery [37]. Likewise, register-based analyses provide system-level insights into trends and outcomes related to surgical safety [43]. Reported hysteroscopic complication rates range from 0.22% to 3.7%, with the lower figures originating from large multicentre and registry datasets such as the Norwegian Gynaecological Endoscopic Registry [44].

Prevention and Management Strategies

Effective prevention and management of life-threatening complications in minimally invasive gynaecology rely on three key pillars: meticulous patient selection, intraoperative vigilance, and rapid multidisciplinary response. Preoperative optimisation of cardiovascular and haematological status reduces the likelihood of intraoperative instability and postoperative intensive-care admission [4,8,10,34]. Continuous monitoring of haemodynamics, oxygenation, and end-tidal CO₂ allows early recognition of concealed haemorrhage, gas embolism, or cardiovascular collapse, facilitating timely escalation [13,14,21].

Anaesthetic strategies tailored to each patient’s physiology, including controlled insufflation pressures and Trendelenburg limits, further mitigate the risk of arrhythmia or circulatory failure [17,35]. Simulation-based multidisciplinary training has proven effective in strengthening team coordination and emergency decision-making during rare but catastrophic events such as vascular injury or massive haemorrhage [10,20,21].

Finally, institutional safety frameworks supported by routine morbidity-and-mortality review, adherence to surgical checklists, and transparent incident reporting create a culture of accountability and continuous improvement, ensuring that early warning signs are acted upon before deterioration necessitates critical-care support [27,28,36].

Outcomes and Future Directions in Patient Safety

When life-threatening complications occur, the outcome is dependent on how fast they are recognised, and a multi-disciplinary approach is taken. Major haemorrhage, visceral trauma, or gas embolism can rapidly evolve into multi-organ failure, necessitating intensive-care support [16,28]. Registry analyses reveal that although mortality is uncommon, morbidity requiring re-operation, prolonged stay, or rehabilitation remains significant [28,37]. Continuous outcome tracking, therefore, underpins both quality improvement and informed consent. Multicentre and registry-based studies confirm that life-threatening events are rare in benign gynaecologic endoscopy but still contribute to a notable proportion of reoperations and extended hospital stays. These datasets offer valuable context for patient counselling and performance benchmarking, underscoring the importance of continued national surveillance [37,43,44].

Within the ICU, comprehensive organ support, antimicrobial therapy, and meticulous fluid-electrolyte management determine survival [10,29]. However, some patients suffer from long-term sequelae such as chronic pain, infertility, and psychological distress, reinforcing the need for structured post-ICU rehabilitation.

Technological advances continue to close remaining safety gaps in minimally invasive gynaecology. Real-time digital monitoring and AI-assisted imaging improve early recognition of physiological deterioration. At the same time, evidence-based simulation and multidisciplinary team-training programmes have been shown to enhance crisis management and reduce severe intraoperative complications [20,42]. Ongoing innovation in surgical instruments and distension systems further supports safer operative environments and decreases systemic risk [7,11,15,34].

At a systemic level, patient safety must transition from an emphasis on the competence of individual practitioners to the accountability of organisations. Integrating predictive analytics and standardised national event reporting can help identify procedural risks early and prevent adverse outcomes [21,28,37]. Structured patient safety monitoring leads to sustained reductions in complication rates when effectively implemented across healthcare networks [37].

Future research should focus on validating AI-driven prediction, refining simulation curricula, and standardising safety metrics. By aligning innovation with robust governance, minimally invasive gynaecology can continue to advance while preserving the highest standards of patient wellbeing.

Limitations and Future Recommendations

This review is constrained by the quality and heterogeneity of the available evidence. The majority of the literature consists of retrospective case series and isolated reports, which limit causal inference and increase the risk of selection and publication bias. True incidence rates of life-threatening complications are almost certainly underestimated because of incomplete reporting and the absence of unified national registries. Furthermore, variable definitions such as those for “major haemorrhage” or “severe organ injury” impede data synthesis and cross-study comparison. Although a structured search strategy was applied, no formal risk-of-bias scoring was undertaken, as the focus was to integrate diverse case-based findings into a pragmatic synthesis. However, this approach may restrict reproducibility and comparability across studies.

Long-term outcomes, including reproductive sequelae, chronic pain, and psychological impact, are poorly studied. These gaps restrict understanding of the full burden of catastrophic complications and limit the development of risk-stratified prevention models.

Future research should focus on developing prospective, multicentre registries that use standardised datasets capturing patient comorbidities, procedural details, fluid or insufflation parameters, complication severity, interventions, and intensive care outcomes. Establishing consistent terminology and international consensus reporting would support reliable benchmarking and comparative analysis.

At the clinical level, embedding simulation-based team training, structured escalation pathways, and digital monitoring into routine practice can enhance early recognition and coordination during emergencies. Technological advances such as predictive analytics, physiological trend monitoring, and AI-driven alert systems represent the next step in improving perioperative safety.

Together, these measures can address ongoing data and safety gaps, ensuring that progress in minimally invasive gynaecology continues to be guided by strong evidence and proactive risk management.

## Conclusions

Life-threatening complications in gynaecological laparoscopy and hysteroscopy remain uncommon but carry profound clinical significance. While minimally invasive techniques have transformed gynaecological surgery by reducing recovery time, postoperative pain, and hospital stay, their potential for catastrophic haemorrhage, visceral injury, gas embolism, or cardiopulmonary collapse necessitates constant vigilance and coordinated multidisciplinary care. Patient comorbidities, anaesthetic management, and timely escalation of care are critical determinants of survival and recovery when deterioration occurs. Continued progress in real-time monitoring, simulation-based team training, and digital risk-prediction tools has strengthened early recognition and response to intraoperative crises. Yet, underreporting, inconsistent terminology, and lack of long-term data continue to obscure the true burden of these events. Sustaining safety in minimally invasive gynaecology, therefore, depends on embedding a proactive safety culture, refining communication between surgical and anaesthetic teams, and reinforcing institutional preparedness to ensure that rare complications never escalate into preventable tragedies.
